# Predicting trajectories of the north star ambulatory assessment total score in Duchenne muscular dystrophy

**DOI:** 10.1371/journal.pone.0325736

**Published:** 2025-06-27

**Authors:** Francesco Muntoni, James Signorovitch, Nathalie Goemans, Adnan Y. Manzur, Nicolae Done, Gautam Sajeev, Jiayang Li, Hanane Akbarnejad, Aarushi Sharma, Susan J. Ward, Erik H. Niks, Laurant Servais, Eugenio Mercuri, Michela Guglieri, Volker Straub, Imelda de Groot, Deborah Ridout, PRO-DMD-01 study investigators, Association Française contre les Myopathies, The NorthStar Clinical Network, Craig McDonald

**Affiliations:** 1 Dubowitz Neuromuscular Centre, NIHR Great Ormond Street Hospital Biomedical Research Centre, Great Ormond Street Institute of Child Health, University College London, & Great Ormond Street Hospital Trust, London, United Kingdom; 2 Analysis Group, Boston, Massachusetts, United States of America; 3 The collaborative Trajectory Analysis Project, Cambridge, Massachusetts, United States of America; 4 Child Neurology, University Hospitals Leuven, Leuven, Belgium; 5 Department of Neurology, Leiden University Medical Center, Leiden, Belgium; 6 Division of Child Neurology, Department of Pediatrics, Centre de Référence des Maladies Neuromusculaires, University Hospital Liège and University of Liège, Liège, Belgium & Department of Paediatrics, MDUK Oxford Neuromuscular Centre & NIHR Oxford Biomedical Research Centre, University of Oxford, Oxford, United Kingdom; 7 Paediatric Neurology, Catholic University, Rome, Italy; 8 Centro Clinico Nemo, Policlinico Gemelli, Fondazione Policlinico Universitario Agostino Gemelli, IRCCS, Rome, Italy; 9 The John Walton Muscular Dystrophy Research Centre, NIHR Newcastle Biomedical Research Centre, Newcastle University and Newcastle Hospitals NHS Foundation Trust, Newcastle upon Tyne, United Kingdom; 10 Academic Medical Center Amsterdam, Rehabilitation Center De Trappenberg in Huizen and Radboud University Medical Center, Nijmegen, Netherlands; 11 Population Policy and Practice Research and Teaching Department, UCL Great Ormond Street Institute of Child Health, London, United Kingdom; 12 Department of Physical Medicine and RehabilitationPediatrics, University of California Davis, Sacramento, California, United States of America; Emory University, UNITED STATES OF AMERICA

## Abstract

The North Star Ambulatory Assessment (NSAA) is a widely used functional endpoint in drug development for ambulatory patients with Duchenne muscular dystrophy (DMD). Accurately predicting NSAA total score trajectories is important for designing randomized trials for novel therapies in DMD and for contextualizing outcomes, especially over longer-term follow-up (>18 months) when placebo-controlled studies are infeasible. We developed a prognostic model for NSAA total score trajectories over at most 5 years of follow-up for patients with DMD aged 4 to <16 years who were initially ambulatory and receiving corticosteroids but no other disease-modifying therapies. The model was based on longitudinal data from four natural history databases: UZ Leuven, PRO-DMD-01 (provided by CureDuchenne), the North Star Clinical Network, and iMDEX. Candidate predictors included age, height, weight, body mass index, steroid type and regime, NSAA total score, rise from floor velocity, and 10-meter walk/run velocity, as well as *DMD* genotype class, index year, and data source. Among N = 416 patients at baseline, mean age was 8.2 years, mean NSAA total score was 24, and 61% were receiving prednisone and 39% deflazacort, with the majority having been treated with daily corticosteroid regimens (69%) relative to other regimens (31%). Patients had an average of four NSAA assessments post-baseline during a median follow-up of 2.6 years (inter-quartile range 1.9 to 3.6 years). The best-fitting model in the full study sample explained 39% of the variation in NSAA total score changes, with prediction errors of ±3.6, 5.1, 5.9, 7.5, 9.5 NSAA units during follow-up years 1–5, respectively. The most important predictors were baseline age, NSAA, rise from floor velocity, and 10-meter walk/run velocity. In conclusion, trajectories of ambulatory motor function in DMD, as measured by the NSAA total score, can be well-predicted using readily available baseline characteristics. We discuss applications of these predictions to DMD drug development.

## Introduction

Duchenne muscular dystrophy (DMD) is a progressive, disabling, and life-limiting disease caused by mutations in the *DMD* gene on the X chromosome, in 15.9 to 19.5 live male births per 100,000 [1]. Without fully functional dystrophin protein, muscles incur cumulative damage, fibrosis, and fatty replacement, leading to progressive weakness and eventual loss of function [2]. Motor function deficits typically present between the ages of 1 and 3 years, and subsequently progress through a series of functional losses [3]. Loss of independent ambulation typically occurs between the ages of 8 and 16 years and is followed by loss of upper-limb function, progressive musculoskeletal deformities, impaired airway clearance, need for mechanical ventilation, cardiomyopathy, and premature death [4,5]. Therapeutic interventions aim to stabilize or slow the disease progression. There is presently no cure for DMD.

While the progression of DMD is inexorable, the rate of progression varies across patients [6,7]. This heterogeneity in progression rates has confounded drug development in DMD and complicates counseling for families. Clinical trials seeking to measure a treatment effect of slowing or stabilizing disease progression must distinguish this effect from the wide range of natural variation, as well as possible impacts of differences in applying standards of care (e.g., age at initiation of steroids, use of daily vs. intermittent regimens, physiotherapy and other factors), which is challenging with the limited sample sizes and follow-up times feasible in DMD clinical studies.

The ability to predict patient outcomes in the absence of an investigational therapy, based on clinical characteristics at baseline, can help address this challenge. Multiple approaches for improving the efficiency of clinical trials, including stratification, enrichment, baseline adjustment and placebo augmentation, as well as contextualizing long-term outcomes from extension studies, depend on such predictions for effectiveness. The more accurately outcomes can be predicted, the more these approaches will improve power and precision for measuring treatment effects [8].

Motor function outcomes in DMD clinical trials and in clinical practice are often measured using the North Star Ambulatory Assessment (NSAA) [9,10]. The NSAA was developed and validated for measuring aspects of motor function important to the lives of patients with DMD [11,12]; it has served as a primary and secondary endpoint in DMD clinical trials [13], and is used in many countries as a routine clinical assessment tool consistent with care guidelines [5].

A wealth of previous research has identified prognostic factors for both 48-week and the longer-term (over 2 years) changes in motor function in DMD, including predictors of changes in NSAA scores [13–17], 6-minute walk distance (6MWD) [18], timed 4 stair climb (4SC) [19], and time to loss of ambulatory function [20,21]. In the present study, we extend this research to develop composite prognostic models for longer-term (up to 5 years) trajectories of NSAA total scores among patients who were initially ambulatory, aged 4 to 16 years, and had already initiated corticosteroid therapy at least 3 months previously. This population was selected to encompass that of many clinical trials in ambulatory DMD.

## Materials and methods

### Data sources

Retrospective clinical data were obtained from four sources: the neuromuscular clinic at Universitaire Ziekenhuizen Leuven (Leuven) from 2011 to 2016 (data accessed for the study on September 25, 2015), the PRO-DMD-01 prospective natural history study (years 2012–2016) [NCT01753804] for which data were provided by CureDuchenne (data accessed for the study on June 7, 2018), a 501(c)(3) DMD patient foundation, the iMDEX natural history study (iMDEX) from 2012 to 2018 [NCT02780492] with data provided by the French Muscular Dystrophy Association (AFM) (data accessed for the study on January 23, 2019), and the North Star UK (NSUK) database from 2005 to 2015 (data accessed for the study on October 4, 2015). Clinical assessments in all data sources were conducted approximately every 6 months. Additional data source characteristics are summarized in S1 Table.

Data collection was approved by the ethics committees from each institution (University Hospitals Leuven, each participating center in iMDEX, PRO-DMD-01 and the North Star Clinical Network). Written informed consent/assent was obtained from each participant or, where appropriate, their caregiver before the study procedures were conducted. Only anonymous, deidentified data were analyzed.

Drawing from these data sources, all patients with a clinic visit meeting the following criteria were included in this study: age at least 4 and under 16 years old, at least minimal ambulatory function (defined as NSAA total score over 5 and 10-meter walk/run [10MWR] under 30s), receiving corticosteroids for at least 3 months, follow-up NSAA assessments available from at least 1 subsequent visit within up to 5 years, and non-missing data on the candidate prognostic factors. The first visit meeting these criteria was designated as the baseline visit.

### Outcome measures

The primary outcome for this study was the trajectory of change in the NSAA total score from baseline to up to 5 years of follow-up. In all contributing data sources, patients’ performance on each of the 17 NSAA activity items was scored by trained clinical staff as either 0 (unable to perform independently), 1 (performs activity using a modified method but is able to complete independently), or 2 (able to perform independently without modification). The NSAA total score is the sum of scores across all activities and ranges from 0 to 34, with higher scores indicating better function [11]. For all data sources, the NSAA was measured by trained assessors, applying the same criteria as used for clinical trials conducted at the centers.

### Candidate predictors

The primary patient characteristics measured at baseline and evaluated for prognostic associations with NSAA outcomes were age, height, weight, body mass index (BMI), steroid type, categorized as receiving prednisone or deflazacort, and baseline measures of motor function available in all data sources: NSAA total score, rise from floor (RFF) velocity, and 10MWR velocity. Sensitivity analyses considered additional factors: steroid regimen (daily vs. other), data source, calendar year, classified according to the distribution of years represented as up to 2009, 2010–2013, 2014, and 2015 and later, consistent with Muntoni et al. 2022 [22], and *DMD* genotype, classified by amenability to certain targeted therapies, as defined in Muntoni et al. 2023 [23].

### Statistical analyses

#### Model development.

Within the development sample, NSAA total score outcomes over up to 5 years post-baseline were studied using generalized estimating equations (GEE) with exchangeable correlation structure. The dependent variable was the change in NSAA total score from baseline to each post-baseline visit. The GEE approach was selected because it is robust to non-Gaussian distributions of NSAA change from baseline. This choice was further supported by our previous analyses using linear mixed effects models [24], which resulted in slightly larger RMSE compared to GEE when validated in independent test data.

A series of models was fitted to the data based on different combinations of predictors, all interacted with time from baseline. Models 1–10 were the primary models investigated (Table 1); Models 11–18 were exploratory (S2 Table). Different shapes for the NSAA total score trajectories over time were evaluated, including linear, quadratic, cubic, and piecewise-linear with knots at 1, 2, 3, and 4 years. Model fit was evaluated by comparing root mean squared errors (RMSE), based on the differences between observed and predicted values across patients and across post-baseline visits. RMSEs were calculated at specific time intervals and averaged across all follow-up time. Predictive accuracy was measured using 5-fold cross-validation. Explained variation in the NSAA total score change was calculated using marginal R-squared. To further characterize prognostic performance, patients were stratified by quartile of predicted 5-year change in NSAA, and the observed NSAA total score trajectories were plotted for patients stratified by quartile.

**Table 1:** Main Model Specifications.

Predictors^a^	Prediction Models									
	M1	M2	M3	M4	M5	M6	M7	M8	M9	M10 (Core)
Linear time	✓									
Quadratic time		✓	✓	✓	✓	✓	✓	✓	✓	✓
Linear age			✓	✓	✓	✓	✓	✓	✓	✓
Quadratic age				✓	✓	✓	✓	✓	✓	✓
NSAA total score					✓			✓	✓	✓
RFF velocity						✓		✓	✓	✓
10MWR velocity							✓	✓	✓	✓
Steroid use									✓	✓
Weight, height, BMI										✓

BMI, body mass index; M, model; NSAA, North Star ambulatory assessment; RFF, rise from floor; 10MWR, 10-meter walk/run.

^a^Check marks indicate that the predictor was included in the model.

### Assessing the impact of missing data

As patients with DMD progress and approach loss of ambulatory function, the choice of whether to conduct the NSAA for an individual who is likely to struggle significantly or to exhibit absence of ambulatory function can vary across care centers. Missing NSAA values may therefore be associated with poor motor function. This type of missing data could bias the observed NSAA data towards better-than-actual function for the studied population. Such bias would affect fitted prediction models as well as any direct analysis of the observed NSAA data.

To understand the magnitude and direction of bias due to missing NSAA assessments, we conducted an imputation analysis to attempt to recover the average NSAA trajectory for the complete data, i.e., the average that would have been observed if no NSAA data were missing. Missing NSAA values were subjected to multiple imputation by chained equations (MICE) under a fully conditional specification using the *mice* package in R [25]. This approach successively imputes missing NSAA values via predictions based on all current and earlier observed and imputed NSAA values and baseline characteristics in the population [25,26]. Additional details are provided in S1 Text. The impact of missing data was assessed by comparing mean NSAA total score trajectories between the observed data and the average across the imputed data sets.

### Sensitivity analysis using machine learning

We evaluated whether a machine learning approach using the Mixed-Effects Random Forest (MERF) model could improve predictive performance compared to GEE [27]. The MERF model incorporated the same predictors as the core GEE model but allowed for more flexible and complex interactions among predictors and non-linear relationships with outcomes. Model hyperparameters were tuned using a cross-validated grid search, as described in S2 Text.

## Results

### Baseline characteristics

**Fig 1:**
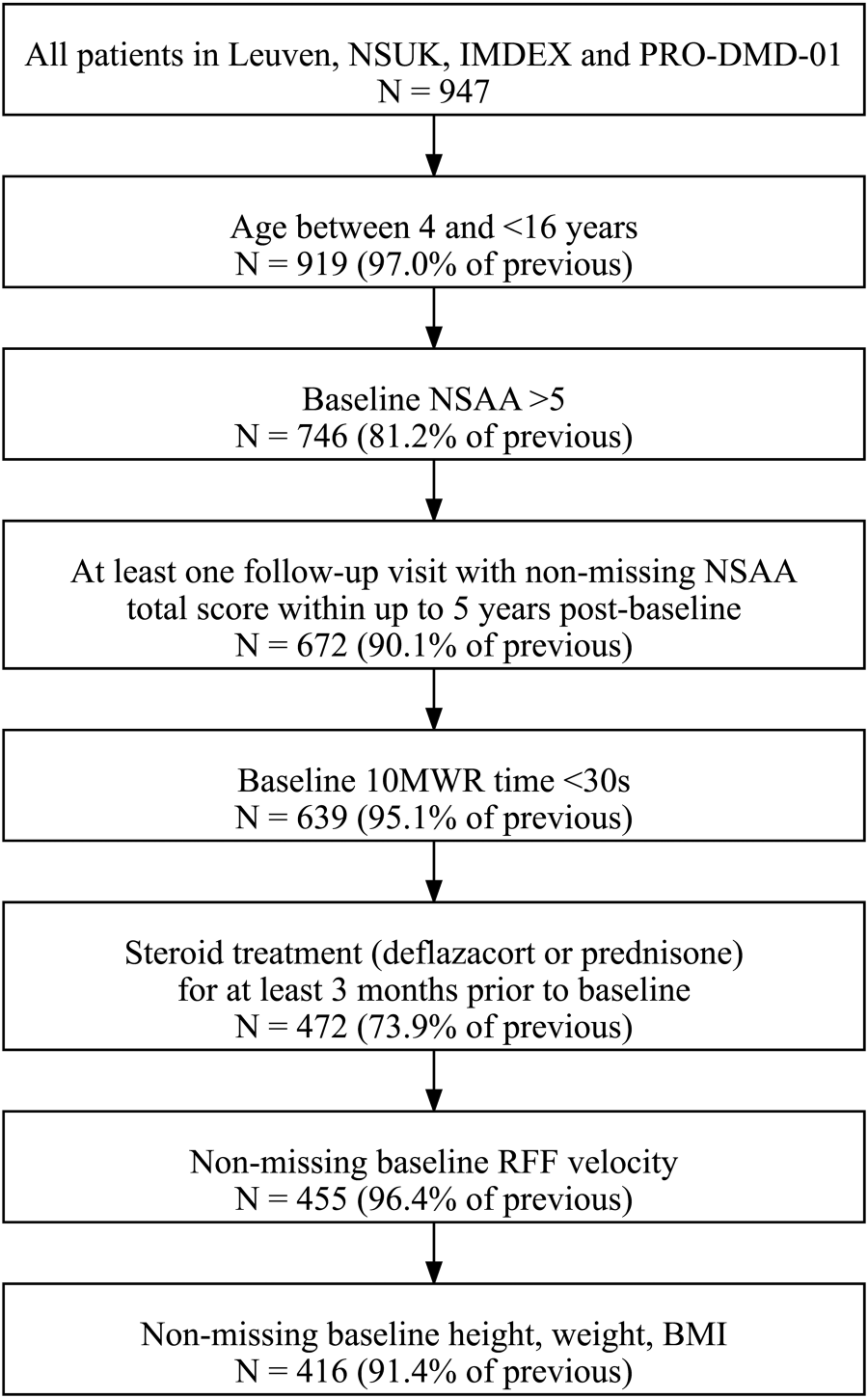
Sample Selection. BMI, body mass index; NSAA, North Star ambulatory assessment; RFF, rise from floor; 10MWR, 10-meter walk/run.

A total of 416 patients and 1,682 post-baseline NSAA assessments were included in the analysis (Fig 1). The majority of the included patients were from the PRO-DMD-01 and NSUK data sources with 174 and 171 patients, respectively (Table 2). Mean ± standard deviation (SD) age at baseline was 8.2 ± 2.4 years. Patients included were ambulatory with a mean NSAA total score of 24.0 ± 6.6 units, a mean ± SD rise from floor (RFF) velocity of 0.2 ± 0.1 1/sec, and a mean ± SD 10MWR of 1.9 ± 0.6 m/sec. Patients had 4 post-baseline NSAA assessments on average (N = 1682 assessments in total), over a median of 2.5 years of follow-up (range 0.3 to 5 years). At baseline, 60.6% and 39.4% were receiving prednisone and deflazacort, respectively. On average, patients were treated with corticosteroids for 25.0 ± 23.0 months before baseline (range 3–116 months), with the majority having been treated with daily corticosteroid regimens (69%) relative to other regimens (31%). The baseline visits for the majority of patients occurred during the years 2010–2015.

**Table 2:** Baseline Characteristics.

	Total	Leuven	iMDEX	NSUK	PRO-DMD-01
**Characteristic** ^a^	**N = 416**	**N = 44**	**N = 27**	**N = 171**	**N = 174**
**Demographics and vitals**					
Age (years)					
Mean ± SD	8.2 ± 2.4	8.7 ± 2.8	7.8 ± 1.9	7.6 ± 2.0	8.8 ± 2.6
Median (range)	7.6 (4.4, 15.5)	8.2 (4.6, 14.6)	7.4 (5.5, 12.7)	7.2 (4.5, 15.3)	8.3 (4.4, 15.5)
Age category (years)					
[4, 5)	12 (2.9%)	2 (4.6%)	0 (0.0%)	7 (4.1%)	3 (1.7%)
[5, 6)	56 (13.5%)	8 (18.2%)	4 (14.8%)	28 (16.4%)	16 (9.2%)
[6, 7)	89 (21.4%)	5 (11.4%)	9 (33.3%)	41 (24.0%)	34 (19.5%)
[7, 8)	70 (16.8%)	7 (15.9%)	3 (11.1%)	31 (18.1%)	29 (16.7%)
[8, 9)	46 (11.1%)	3 (6.8%)	4 (14.8%)	22 (12.9%)	17 (9.8%)
[9, 10)	50 (12.0%)	3 (6.8%)	4 (14.8%)	19 (11.1%)	24 (13.8%)
[10, 11)	34 (8.2%)	3 (6.8%)	1 (3.7%)	13 (7.6%)	17 (9.8%)
[11, 12)	24 (5.8%)	8 (18.2%)	1 (3.7%)	5 (2.9%)	10 (5.8%)
[12, 13)	13 (3.1%)	3 (6.8%)	1 (3.7%)	2 (1.2%)	7 (4.0%)
[13, 14)	13 (3.1%)	0 (0.0%)	0 (0.0%)	2 (1.2%)	11 (6.3%)
[14, 15)	6 (1.4%)	2 (4.6%)	0 (0.0%)	0 (0.0%)	4 (2.3%)
[15, 16)	3 (0.7%)	0 (0.0%)	0 (0.0%)	1 (0.6%)	2 (1.2%)
Height (cm)					
Mean ± SD	120.4 ± 11.6	119.3 ± 12.4	119.9 ± 11.0	119.4 ± 10.9	121.7 ± 12.1
Median (range)	119.2 (94.5, 156.9)	119.2 (95.0, 151.6)	119 (104.0, 144.0)	117 (97.5, 156.9)	121.4 (94.5, 152.0)
Weight (kg)					
Mean ± SD	27.7 ± 9.6	28.8 ± 11.4	26.3 ± 8.6	27.2 ± 9.1	28.1 ± 9.9
Median (range)	24.7 (14.2, 70.4)	24.6 (14.2, 60.2)	24.7 (17.0, 48.7)	24 (16.1, 59.5)	25.2 (14.7, 70.4)
BMI (kg/m^2^)					
Mean ± SD	18.6 ± 3.5	19.5 ± 4.3	17.8 ± 2.8	18.6 ± 3.2	18.5 ± 3.7
Median (range)	17.5 (8.8, 31.5)	17.5 (14.5, 30.5)	17.3 (13.8, 25.8)	17.6 (12.7, 30.8)	17.5 (8.8, 31.5)
**Steroid use**					
Steroid type					
Prednisone	252 (60.6%)	5 (11.4%)	21 (77.8%)	163 (95.3%)	63 (36.2%)
Deflazacort	164 (39.4%)	39 (88.6%)	6 (22.2%)	8 (4.7%)	111 (63.8%)
Steroid duration (months)					
Mean ± SD	24.9 ± 23.0	30.2 ± 24.5	–	17.1 ± 15.4	31.3 ± 26.3
Median (range)	17.51 (3.0, 116.0)	26.0 (3.2, 76.4)	–	12 (3.0, 80.0)	24.4 (3.1, 116.0)
Steroid regimen					
Daily	280 (69.0%)	44 (100.0%)	18 (72.0%)	88 (54.0%)	130 (74.7%)
Non-daily	126 (31.0%)	0 (0.0%)	7 (28.0%)	75 (46.0%)	44 (25.3%)
**Ambulatory motor function**					
NSAA total score					
Mean ± SD	24.0 ± 6.6	22.9 ± 7.2	24.7 ± 6.5	24.6 ± 6.0	23.5 ± 7.0
Median (range)	25 (6.0, 34.0)	25 (9.0, 33.0)	24 (11.0, 34.0)	25 (10.0, 34.0)	25 (6.0, 34.0)
RFF (velocity) (1/sec)					
Mean ± SD	0.2 ± 0.1	0.2 ± 0.1	0.2 ± 0.1	0.2 ± 0.1	0.2 ± 0.1
Median (range)	0.2 (0.0, 0.7)	0.2 (0.0, 0.4)	0.2 (0.1, 0.4)	0.2 (0.0, 0.7)	0.2 (0.0, 0.6)
10MWR (velocity) (m/sec)					
Mean ± SD	1.9 ± 0.6	2.0 ± 0.7	1.9 ± 0.5	1.7 ± 0.6	1.9 ± 0.5
Median (range)	1.8 (0.5, 4.1)	2.0 (0.6, 4.1)	2.0 (1.2, 2.8)	1.7 (0.7, 4.0)	1.9 (0.5, 3.3)
**Year, genotype classes and follow-up duration**					
Year category					
Up to 2009	144 (34.7%)	20 (45.5%)	0 (0.0%)	124 (72.9%)	0 (0.0%)
2010–2013	196 (47.2%)	20 (45.5%)	15 (55.6%)	46 (27.1%)	115 (66.1%)
2014	67 (16.1%)	3 (6.8%)	5 (18.5%)	0 (0.0%)	59 (33.9%)
2015 and later	8 (1.9%)	1 (2.3%)	7 (25.9%)	0 (0.0%)	0 (0.0%)
Genotype classification					
Exon 44 skip	51 (12.6%)	3 (7.0%)	11 (40.7%)	15 (9.4%)	22 (12.6%)
Exon 45 skip	55 (13.6%)	5 (11.6%)	5 (18.5%)	14 (8.8%)	31 (17.8%)
Exon 51 skip	36 (8.9%)	4 (9.3%)	1 (3.7%)	22 (13.8%)	9 (5.2%)
Exon 53 skip	45 (11.1%)	3 (7.0%)	4 (14.8%)	12 (7.5%)	26 (14.9%)
Other skip-amenable	49 (12.1%)	6 (14.0%)	4 (14.8%)	16 (10.0%)	23 (13.2%)
Nonsense	22 (5.4%)	2 (4.6%)	0 (0.0%)	1 (0.63%)	19 (10.9%)
All others	146 (36.1%)	20 (46.5%)	2 (7.4%)	80 (50.0%)	44 (25.3%)
Follow-up duration (years)					
Mean ± SD	2.6 ± 1.2	3.2 ± 1.5	2.4 ± 1.5	3.0 ± 1.3	2.2 ± 0.7
Median (range)	2.5 (0.3, 5.0)	3.8 (0.5, 5.0)	2.4 (0.4, 4.7)	3.1 (0.3, 5.0)	2.2 (0.4, 3.3)

BMI, body mass index; NSAA, North Star ambulatory assessment; RFF, rise from floor; SD, standard deviation; 10MWR, 10-meter walk/run.

^a^Counts and percentages are presented for categorical characteristics, unless otherwise noted.

### Prognostic models

**Fig 2:**
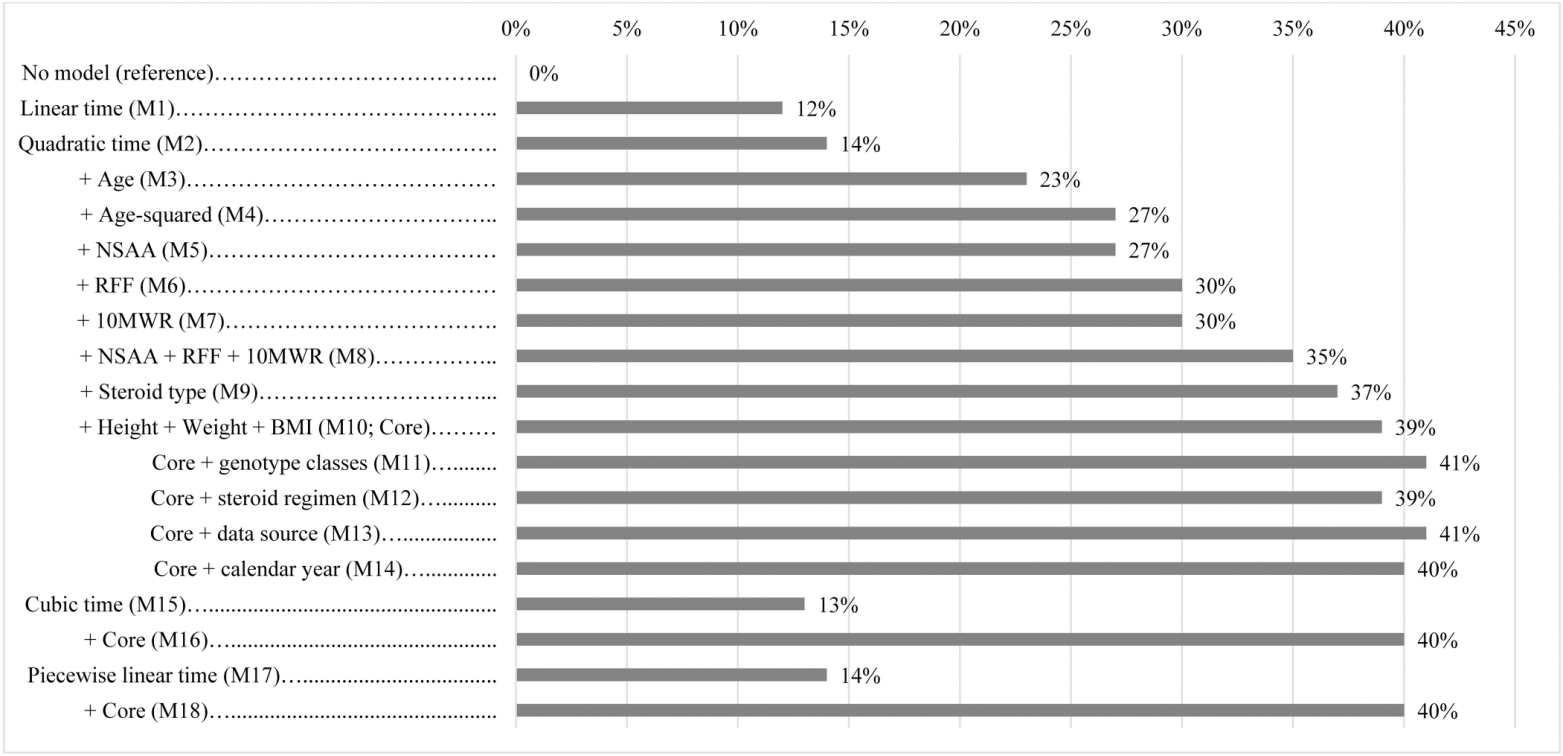
Percentages of Variation in NSAA Total Score Outcomes (Marginal R^2^) Explained by Models Incorporating Different Factors. BMI, body mass index; NSAA, North Star ambulatory assessment; M, model; RFF, rise from floor; 10MWR, ten-meter walk/run. ^a^ Percentage of explained variation was measured using marginal R^2^. ^b^ Piecewise linear time (M11) includes knots at years 1, 2, 3, and 4.

Sequential addition of baseline characteristics identified age, age squared, anthropometric measures (i.e., height, weight, and BMI), steroid type, and motor function measures (i.e., NSAA, RFF and 10MWR), along with quadratic effects of time, as together explaining 39% of the variability in NSAA outcomes (Fig 2). Age alone (modeled as the effects of age and age squared) explained 27% of the variability in NSAA outcomes. No single functional measure increased this beyond 30%, but the addition of all 3 functional measures together increased the explained variation to 35%, with an additional 2% explained by including steroid type and a further 2% explained by including body size, to reach 39%. When subjected to cross-validation, the model including all of these characteristics, Model 10, performed best, by having the lowest RMSE overall and over each year of follow-up relative to Models 1–9 (Table 3).

**Table 3:** Main Models Predictive Performance.

Model^a^	Time from Baseline											
	1-year (N = 379)^b^		2-year (N = 327)^b^		3-year (N = 214)^b^		4-year (N = 115)^b^		5-year (N = 50)^b^		Total (N = 416)^b^	
	RMSE	CV-RMSE	RMSE	CV-RMSE	RMSE	CV-RMSE	RMSE	CV-RMSE	RMSE	CV-RMSE	RMSE	CV-RMSE
M1	3.98	3.99	5.82	5.85	7.30	7.31	9.12	9.09	9.32	9.38	6.24	6.27
M2	3.94	3.94	5.80	5.84	7.18	7.18	9.02	8.99	9.46	9.51	6.19	6.22
M3	3.72	3.74	5.53	5.56	6.67	6.70	8.26	8.25	9.37	9.48	5.83	5.87
M4	3.69	3.69	5.34	5.39	6.47	6.54	8.10	8.07	9.16	9.43	5.68	5.76
M5	3.70	3.72	5.35	5.42	6.49	6.60	8.13	8.16	9.15	9.51	5.70	5.81
M6	3.67	3.68	5.21	5.29	6.19	6.26	8.12	8.07	9.11	9.51	5.57	5.67
M7	3.65	3.66	5.25	5.30	6.30	6.38	7.81	7.90	9.24	9.86	5.58	5.69
M8	3.57	3.60	5.08	5.19	5.94	6.11	7.61	7.79	9.01	9.76	5.38	5.55
M9	3.56	3.59	5.06	5.17	5.80	6.00	7.34	7.52	8.96	9.77	5.30	5.49
M10 (Core)	3.55	3.58	4.96	5.09	5.69	5.91	7.21	7.47	8.61	9.53	5.20	5.43

CV-RMSE, cross-validated root mean squared error; M, model; RMSE, root mean squared error.

^a^ Predictors included in each model can be found in Table 1.

^b^ “N” corresponds to the number of patients with available data at each time point.

The prognostic impact of adding other patient characteristics to the best-performing model, Model 10, was assessed, with sample sizes declining in some cases due to missing baseline data. Genotype class (n = 404 patients; n = 1645 assessments), daily vs. other steroid regimen (n = 406 patients; n = 1645 assessments), and calendar year (n = 415; n = 1678 assessments), as well as more complex models for the shape of NSAA total score change trajectories over time, did not substantially improve predictions. None of these additional predictors improved explained variation by more than 2% and cross-validated prediction errors generally worsened or improved very slightly in a few instances (S3 Table).

Considering the predictive performance, data availability, and knowledge that true trajectories are non-linear over longer periods, Model 10 was selected as the core model. Estimated coefficients for Model 10 are summarized in Table 4. Estimated coefficients for models incorporating steroid regimen or genotype class are included in S4 Table.

**Table 4:** Fitted Prediction Model for NSAA Trajectory.

Baseline characteristics^a^	Coefficient	Standard Error	P-value
Linear time × linear age	−2.265	0.952	0.017
Linear time × quadratic age	0.102	0.053	0.054
Quadratic time × linear age	0.178	0.306	0.561
Quadratic time × quadratic age	−0.008	0.018	0.670
Linear time × NSAA	−0.182	0.072	0.012
Quadratic time × NSAA	0.029	0.023	0.198
Linear time × velocity RFF	13.91	3.487	0.000
Quadratic time × velocity RFF	−2.101	1.013	0.038
Linear time × velocity 10MWR	1.811	0.789	0.022
Quadratic time × velocity 10MWR	−0.34	0.24	0.156
Linear time × steroid use	−1.087	0.713	0.127
Quadratic time × steroid use	−0.06	0.258	0.816
Linear time × height	−0.307	0.131	0.019
Quadratic time × height	0.029	0.044	0.514
Linear time × weight	0.501	0.27	0.064
Quadratic time × weight	−0.027	0.09	0.760
Linear time × BMI	−0.701	0.427	0.100
Quadratic time × BMI	0.046	0.131	0.726

BMI, body mass index; NSAA, North Star ambulatory assessment; RFF, rise from floor; 10MWR, 10-meter walk/run.

^a^Time and age are measured in years; RFF velocity is measured as 1/ second; 10MWR velocity is measured as meters/ second; height is measured in cm; weight is measured in kg; BMI is measured in kg/m^2^

**Fig 3:**
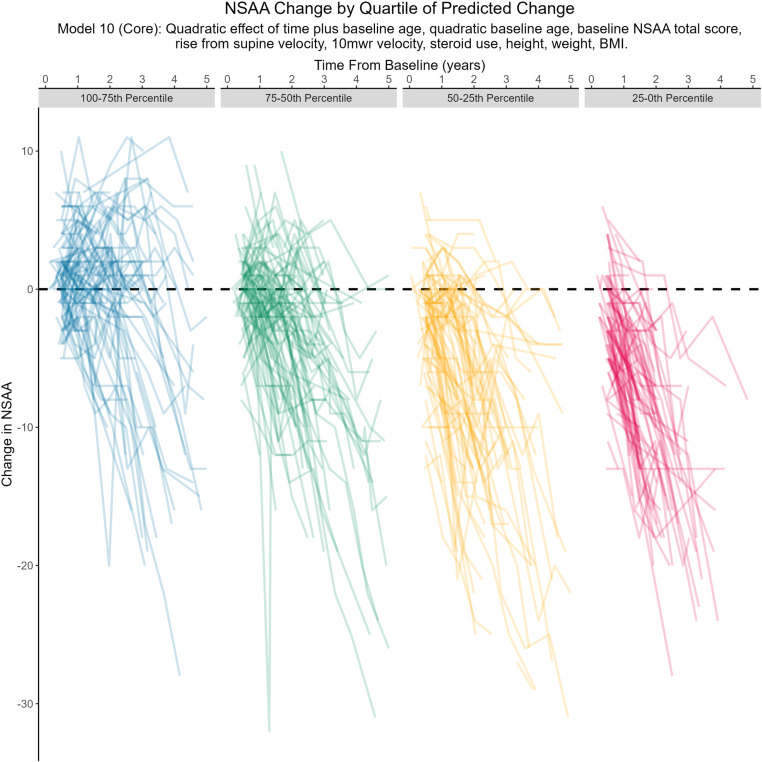
Observed NSAA Total Score Changes by Quartile of Predicted Change (Core Model). NSAA, North Star ambulatory assessment.

When the observed NSAA total score change trajectories were stratified by quartiles of predicted 1-year change based on Model 10 (Fig 3), the first quartile (0–25^th^ percentile) included more patients with rapid declines, the second and third quartiles included successively fewer rapid declines, and more patients with stable or moderate declines; the fourth quartile included more patients with stable or improving NSAA total scores. Visually, the variability in NSAA total score changes remained high across groups; all groups included patients with improvements exceeding 5 units and declines exceeding 5 units.

### Assessment of missing data

The proportion of patients with missing NSAA data increased over follow-up time, with missing proportions of 24%, 47%, 73%, 88%, and 92% observed at months 12, 24, 36, 48, and 60, respectively. The majority of missingness was monotone rather than intermittent, i.e., due to dropout and/or cessation of NSAA assessment for the remainder of the patients’ follow-up rather than sporadic missing assessments that are both preceded and followed by completed assessments (S1 Fig).

**Fig 4:**
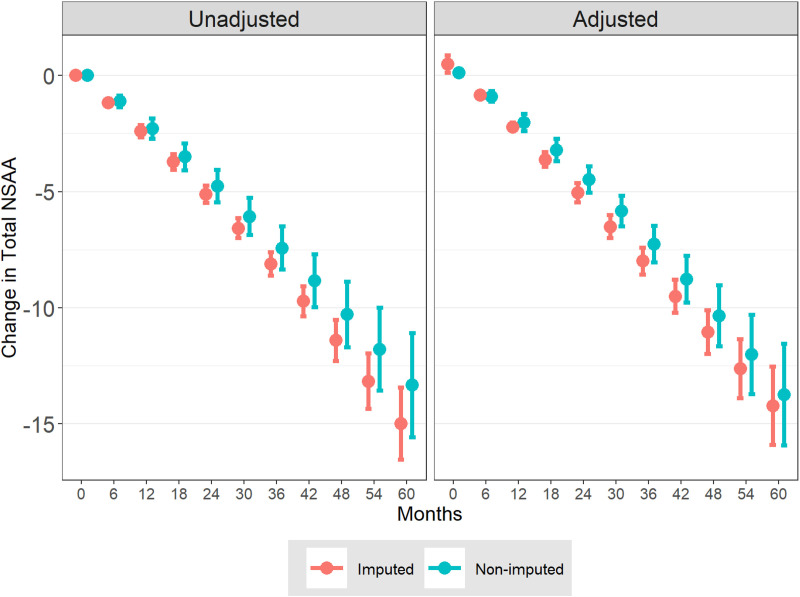
Average NSAA Total Score Trajectories with and without Imputation of Missing NSAA Data. Abbreviations: BMI, body mass index; NSAA, North Star ambulatory assessment; RFF, rise from floor; 10MWR, 10-meter walk/run. **Notes:** Adjusted changes were estimated from generalized estimating equation models controlling for baseline covariates including age, NSAA total score, 10MWR, RFF, steroid type, height, weight, and BMI. Error bars represent 95% confidence intervals for the mean change in NSAA total score at each timepoint.

Multiple imputation of these missing NSAA values resulted in longer periods with low NSAA total scores (S2 Fig) and consequently greater average declines in NSAA total scores after imputation compared with the observed NSAA data alone (Fig 4). This pattern was observed with and without adjustments for baseline covariates (Fig 4). Inferred bias in the overall population’s mean NSAA trajectory due to missing data increased over time, while remaining small in magnitude for a centrally representative trajectory, from +0.1 units at year 1 to +1.7 units by year 5 (Table 5). Adjustment for baseline covariates further decreased the inferred bias during years 4 and 5 (Table 5). While the bias due to missing NSAA data was small on average, further investigation of subpopulations indicated greater bias for patients aged 10 years or older at baseline. In this group the estimated bias due to missing data became larger at 3 years of follow-up and later, reaching over 5 units by year 5 (S3 Fig). Bias remained small and positive for patients with baseline age younger than 10 years, or with baseline NSAA total score above or below the median (S4-S6 Figs).

**Table 5:** Estimated Mean Changes in NSAA Total Score with and without Accounting for Missing NSAA Data, and Adjustment for Baseline Covariates.

	Change in NSAA Total Score (Mean ± SE)				
	1-year	2-year	3-year	4-year	5-year
Non-imputed unadjusted	−2.3 ± 0.2	−4.8 ± 0.4	−7.4 ± 0.5	−10.3 ± 0.7	−13.3 ± 1.1
Imputed unadjusted	−2.4 ± 0.1	−5.1 ± 0.2	−8.1 ± 0.3	−11.4 ± 0.5	−15.0 ± 0.8
Non-imputed adjusted^a^	−2.0 ± 0.2	−4.5 ± 0.3	−7.3 ± 0.4	−10.3 ± 0.7	−13.7 ± 1.1
Imputed adjusted^a^	−2.2 ± 0.1	−5.1 ± 0.2	−8.0 ± 0.3	−11.1 ± 0.5	−14.2 ± 0.9

Abbreviations: BMI = body mass index; NSAA = North Star Ambulatory Assessment; RFF = rise from floor; SE = standard error; 10MWR = 10-meter walk/run.

Note:

^a^Adjustment was conducted for baseline covariates including age, NSAA total score, 10MWR, RFF, steroid type, height, weight, and BMI.

### Machine learning sensitivity analysis

When comparing the predictive performance of the GEE and MERF approaches, we found that the tuned MERF model performed slightly worse than the core GEE model over the first 4 years of follow-up, with cross-validated RMSE values consistently higher for MERF. While MERF performed numerically better at year 5 (RMSE: 9.49 vs. 9.53), this difference was not meaningful or statistically significant as 95% confidence intervals overlapped substantially. For both models, prediction errors increased in later years, reflecting increased prediction uncertainty and data sparsity with longer follow-up (S7 Fig).

## Discussion

Average NSAA total score trajectories were well-predicted over up to 5 years using a patient’s baseline age, NSAA total score, timed 10MWR, timed RFF, corticosteroid type, height, weight, and BMI to an accuracy of 3.6, 5.1, 5.9, 7.5 and 9.5 NSAA units at years 1–5, respectively; predictions for group means had greater precision, with standard errors of 0.2, 0.3, 0.4, 0.7, and 1.1, respectively. This level of predictive accuracy is suitable for many applications in drug development, such as those summarized in Table 6. Since these predictions are based on more than 400 subjects, they exhibit lower variability than the smaller samples, generally fewer than 100 subjects, enrolled in DMD clinical trials. Such predictions can serve to benchmark or contextualize NSAA outcomes in clinical trials, including open-label extension studies, beyond the duration of follow-up for which placebo controls would be feasible in ambulatory DMD. As the predictive accuracy of this model decreases over time, treatment effects would need to be larger at later time points to be distinguishable from predictive controls with high confidence.

**Table 6:** Use Cases for Prognostic Factors and Prognostic Models in Drug Development.

Application of prognostic factors or models in drug development	Considerations for use
Inclusion/exclusion criteria, enrichment	Enrichment for modifiable trajectories can improve power, and depends on the ability to predict trajectories. The better the prediction the better the enrichment that can be achieved.
Stratification of randomization	The purpose of stratified randomization is to ensure balance on important prognostic factors. The logistical complexity of stratification is best invested in the most important prognostic factors.
Adjustment for baseline characteristics	Adjustment for prognostic factors improves precision and power by reducing unexplained variation. Combining prognostic factors into a composite prognostic score is more efficient than adjusting for many factors separately. When pre-specified and applied to randomized trials this approach can improve power without threatening trial validity or protection of Type I error [28].
External controls, placebo augmentation	When using external data to measure drug effects in a clinical trials, with a stand-alone external control or placebo augmentation, adjustment for baseline prognostic factors is critical to reduce risk of confounding bias [29]. This can be accomplished by adjusting for or matching on known prognostic factors, a prognostic score (i.e., a prediction of each individual’s outcome given their characteristics) or by directly comparing treated patient outcomes to predicted controls based on a validated prediction model.

Important predictors of longer-term NSAA outcomes included those identified in prior studies of 48-week change in the NSAA total score [22], 6MWD [18], and 4SC [19], including multiple measures of baseline function, in this case baseline NSAA, RFF, and 10MWR, along with corticosteroid type, height, weight and BMI [30]. One exception was that age was identified as an important predictor of longer-term NSAA outcomes in the present study, whereas age was not strongly predictive of 48-week outcomes in multiple prior studies with large sample sizes [18,19,22,30]. This may be due to the fact that our prognostic model focuses on long-term NSAA outcomes (up to 5 years), whereas these prior studies examined shorter-term predictions. The impact of age may become more pronounced over longer periods.

It was initially surprising that steroid regimen (daily vs. other) did not add substantial prognostic value in the present study, especially in light of daily regimens showing better motor function outcomes over 3 years, regardless of steroids type, compared with intermittent prednisone in the randomized FOR-DMD trial [31]. These findings are compatible, considering that FOR-DMD measured the *effect* of assigned steroid regimen among *steroid naïve patients*, whereas the present study assessed the *predictive value* of steroid regimen among patients *already receiving* steroids, with the average patient having initiated steroids over 2 years before baseline. The predictive values of both steroid type and regimen were small in the present study, explaining less than 2% of the variation in NSAA total score outcomes, after accounting for the predictive value of baseline age and motor function profile. The future NSAA trajectory of a patient who has already been receiving steroid treatment would be better predicted by his current age and functional profile than by steroid type or regimen. Indeed, baseline functional profiles in the present study already reflect effects of pre-baseline steroid type and regimen, leaving less room for those factors to add predictive value going forward. Therefore, predictive values in the present study should not be interpreted causally.

While real-world data are important for understanding disease progression, missing assessments of ambulatory motor function have been difficult to avoid in large, long-term, real-world databases in DMD, especially as patients approach loss of ambulatory function. Our analyses indicated that the bias due to missing NSAA assessments is small in magnitude and, importantly, positive in direction. That is, reported NSAA data present a more favorable picture of the natural history of disease, on average, than the reality experienced by patients with DMD, since the missing NSAA assessments tend to contain lower scores than the observed assessments. This direction of bias would render external controls based on these data sources conservative with respect to missing NSAA data, with bias towards smaller-than-actual treatment effects in comparative analyses, regardless of the analysis method used (e.g., predicted controls based on the current model, or direct use of the patient-level natural history data via multivariable regression, matching approaches, or placebo augmentation). A straightforward and conservative analysis of the observed NSAA data could be supplemented by multiple imputation to account for this bias.

Other models for NSAA outcomes have been developed in DMD. The Duchenne Regulatory Science Consortium (D-RSC) base model includes baseline NSAA, age corticosteroid use (use vs. naïve), and genotypes as predictors [32], and is designed to simulate a distribution of NSAA trajectories that can be used to inform clinical trial simulation and design. This model used a smaller set of predictors, and a different mathematical approach commonly used in pharmacometrics (a Chapman-Richards model), whereas the present study used a broader set of baseline predictors, combining multiple measures of motor function as well as methods commonly used in analyses of efficacy outcomes in cohort studies and clinical trials. Other previous studies focused on 3-year NSAA changes found the effect of age, baseline 6MWT, and steroid treatment to be significant on the disease progression [17]. Studies also showed that there is a significant difference in NSAA trajectories over 3 years based on mutation subgroups [15]. Similar findings have been found for 6MWT, which emphasizes the importance of considering longer-term projections for this outcome as well [14,17]. The impact of the *DMD* genotype classes studied here was previously found to be small for 1-year changes in NSAA and for times from baseline to reaching 10MWR over 10 seconds, especially after adjusting for a patient’s baseline motor function status [33].

The five-year follow-up horizon was selected as it represents the longest period reasonably supported by the data while maintaining sufficient sample size for reliable estimation. Our model extends beyond the typical 48-week to 18-month duration of placebo-controlled trials, addressing the need for longer-term outcome predictions while remaining applicable to shorter timeframes as well. The focus on ambulatory patients aged 4 to under 16 years receiving corticosteroids was chosen to reflect typical enrollment criteria for contemporary DMD clinical trials, as the NSAA can only be meaningfully measured in ambulatory patients and corticosteroid therapy is now standard of care. This alignment with current trial populations increases the model’s direct applicability for trial design and interpretation in the modern treatment landscape.

### Limitations

This study has several limitations. First, some predictors, including 4SC and 6MWD, were not available in all data sources and were therefore not evaluated. Other predictors, including magnetic resonance imaging (MRI) metrics and genetic modifiers, were not available and should also be investigated. Additional *DMD* genotypes, beyond the classes studied here, were small in number and were not studied. Some of the covariates used in the exploratory models, such as genotype class and corticosteroid regimen, included missing data, leading to reduced sample size. The quality of non-missing data is also uncertain in some of these factors, e.g., steroid frequency and steroid dose per kg bodyweight are not regularly updated in all databases and must be carried forward over long periods. Future research with more detailed medication data would be valuable to explore how real-world steroid dose relative to target may impact NSAA trajectories. The majority of patients in the present study self-reported their race as White, or did not have race recorded. The impact of patient race as a prognostic factor, or as a modifier of other factors, was not studied. Moreover, only a fraction of the included patients (approximately 12%) have data up to 5 years of follow-up in this study, which may affect both the precision and generalizability of the model predictions at year 5.

The present model includes linear and quadratic effects of time on the NSAA trajectory, which are modified by the included baseline characteristics. While it is possible that different modeling assumptions (e.g., functional forms for NSAA trajectories or impacts of predictors, such as interactions or threshold effects) could improve prediction, this seems unlikely with the present data given the lack of improvement seen with the flexible machine learning approach, which explored a diverse multitude of possible models.

The present study does not explore the strengths and limitations of different ways of applying prediction models to use cases in drug development. Many of these use cases warrant caution and carry risks of bias that have been extensively described [29,34]. In particular, while 1-year changes in the NSAA total score have been found to be highly consistent across data sources, natural history studies and clinical trial placebo arms, geographies, and years 2003–2016 [22], there is still a risk of “open-label bias.” This can occur when performance outcomes such as the NSAA are compared between patients receiving open-label investigational therapy and external or predicted controls. The concern is that patients, caregivers, or clinical assessors might record better performance, even unintentionally, when the patient is known to be on the investigational therapy due to greater motivation, hope for improvement, or other factors. Given the progressive nature of DMD there is a limit on how much a patient could over-perform on NSAA due to such biases, especially over multi-year time periods. Better quantifying the risk and magnitude of open-label bias in NSAA and other functional outcomes in DMD represents an important step in the use and interpretation of external controls.

## Conclusions

Prognostic models for changes in NSAA over 5 years are feasible in DMD. Additional research is warranted to ensure that predictions are accurate, and that validated models apply across broad populations with DMD. The prediction model developed in the present study is faithful to the studied data sources and can provide reasonable predicted controls to help contextualize treated patients over up to 5 years of follow-up.

## Supporting information

S1 Text Multiple Imputation. (DOCX)

S2 Text Methods for MERF hyperparameter tuning. (DOCX)

S1 Fig Patterns of Missingness in NSAA Total Score Data over Time. NSAA, North Star ambulatory assessment. NSAA assessments were assigned to the closest 6-month time point, drawing from the closest measured value within ±3 months of each time point. Each row represents an individual patient.
(TIF)

S2 Fig Trajectories of NSAA Total Score vs. Age with vs. without Imputation of Missing NSAA Values. NSAA, North Star ambulatory assessment.
(TIF)

S1 Table Data Source Characteristics. (DOCX)

S2 Table Exploratory Model Specifications. (DOCX)

S3 Table Exploratory Model Predictive Performance. (DOCX)

S4 Table Fitted Models Including Steroid Regimen (Daily vs. Other) and Genotype Class. (DOCX)

S3 Fig Predicted Change in Imputed and Unimputed NSAA Total Score Up to 60 Months Post-Baseline Baseline among Patients with Baseline Age < 10 Years. (TIF)

S4 Fig Predicted Change in Imputed and Unimputed NSAA Total Score Up to 60 Months Post-Baseline Baseline among Patients with Baseline Age ≥ 10 years. (TIF)

S5 Fig Predicted Change in Imputed and Unimputed NSAA Total Score Up to 60 Months Post-Baseline Baseline among Patients with Baseline NSAA ≥ Median (25 Units). (TIF)

S6 Fig Predicted Change in Imputed and Unimputed NSAA Total Score Up to 60 Months Post-Baseline Baseline among Patients with Baseline NSAA < Median (25 Units). (TIF)

S7 Fig Cross-validated RMSE (95% CIs) of the MERF and Core GEE Models, by Follow-up Year and Overall. (TIF)
