# Supplementary material for: Predicting trajectories of the north star ambulatory assessment total score in Duchenne muscular dystrophy
Source: PLoS One. 2025 Jun 27;20(6):e0325736. doi: 10.1371/journal.pone.0325736 (PMC12204569; doi:10.1371/journal.pone.0325736)
Supplement: S1 Text — (DOCX) [file pone.0325736.s001.docx]

**S1 Text. Multiple Imputation.**

***Methods detail for multiple imputation***

For the multiple imputation analysis, all post-baseline NSAA assessments were aggregated to equally-spaced 6-month time points from month 6 to month 60 post-baseline, drawing from the closest measured value within ±3 months of each time point. Time points without a close-enough observed NSAA value were considered to have missing NSAA. Missing NSAA values were then subjected to multiple imputation by chained equations (MICE) under a fully conditional specification. This approach imputes missing NSAA values via predictions based on all current and earlier observed and imputed NSAA values and baseline characteristics in the population, including age, RFF velocity, 10MWR velocity, steroid use, weight, height, and BMI (21, 22). The MICE approach assumes that data are missing-at-random, i.e., that missingness is independent of the underlying NSAA values themselves, conditional on all other observed data. Imputed values were based on predictive mean matching to ensure suitable imputed values (i.e., whole numbers between 0 and 34) (31, 32). After MICE, if a patient had imputed value of 0 at a post-follow-up visit followed by an increased NSAA score, those values were truncated to 0. However, observed values were kept the same and not truncated to 0 even if the value was greater than 0. A total of 100 imputations, each consisting of 30 MICE iterations of model fitting and predictive imputation, were conducted. Model estimates were then pooled using Rubin’s approach to estimate the mean trajectory and associated standard errors under multiple imputation (33). Trace plots were inspected to confirm convergence. Imputations were conducted using the *mice* package in R (21).

***Impacts of multiple imputation by subpopulation***

In addition to the overall impacts of multiple imputation reported in the main body of the manuscript, differences between imputed and non-imputed NSAA assessments were studied by subpopulation to further assess the risk bias due to missing data. Among patients aged < 10 years at baseline, differences between mean NSAA trajectories with imputed vs. non-imputed values were small (1-2 units) during all 5 years of follow-up, with the observed, non-imputed values indicating bias towards better preservation of NSAA function over time (S3 Fig).

In contrast, for patients aged >= 10 years at baseline, the difference between the imputed vs. non-imputed analyses becomes substantially larger at 3 years of follow-up and later, reaching over 5 units by year 5 (S4 Fig). The observed natural history data are biased towards showing greater preservation of NSAA, especially among older patients followed beyond 3 years, than the true level of NSAA change that would have been observed if all patients were consistently assessed for NSAA during follow-up. The substantial increase in bias with longer follow-up coincides with a substantial drop in sample size. There are n=92 patients with baseline age >= 10 years. Numbers with NSAA assessments at 12, 24, 36, 48 and 60 months, respectively, are 62, 37, 16, 8 and 4. Thus, predictions beyond year 3 for patients aged >= 10 years at baseline are informed by no more than 16 subjects with observations directly relevant to this group.

When subjects were stratified by baseline NSAA, based on the median of > 25 units, both groups showed small levels of bias towards greater preservation of NSAA total scores (S5 and S6 Figs).
