# Supplementary material for: Predicting trajectories of the north star ambulatory assessment total score in Duchenne muscular dystrophy
Source: PLoS One. 2025 Jun 27;20(6):e0325736. doi: 10.1371/journal.pone.0325736 (PMC12204569; doi:10.1371/journal.pone.0325736)
Supplement: S2 Text — (DOCX) [file pone.0325736.s002.docx]

**S2 Text. Methods for MERF hyperparameter tuning.**

To optimize the MERF model performance, we conducted a comprehensive two-stage cross-validated grid search for hyperparameter tuning. Cross-validation was employed at each step to ensure the selected parameters would generalize well to unseen data. In the first stage, we performed a coarse grid search exploring a wide range of values for key hyperparameters: mtry (number of variables randomly sampled as candidates at each split) ranging from 4 to 12 by increments of 2, and ntree (number of trees to grow in the forest) ranging from 300 to 600 by increments of 100. Based on cross-validated RMSE results, we identified promising regions for a more focused search. In the second stage, we conducted a fine-tuned grid search with mtry values from 8 to 12 by increments of 1 and ntree values from 450 to 550 by increments of 25. The final tuned MERF model used mtry=10 and ntree=450, which provided the lowest cross-validated prediction error. This tuning process guarded against overfitting of the MERF model.
