# Supplementary figures and images for: Predicting trajectories of the north star ambulatory assessment total score in Duchenne muscular dystrophy
Source: PLoS One. 2025 Jun 27;20(6):e0325736. doi: 10.1371/journal.pone.0325736 (PMC12204569; doi:10.1371/journal.pone.0325736)

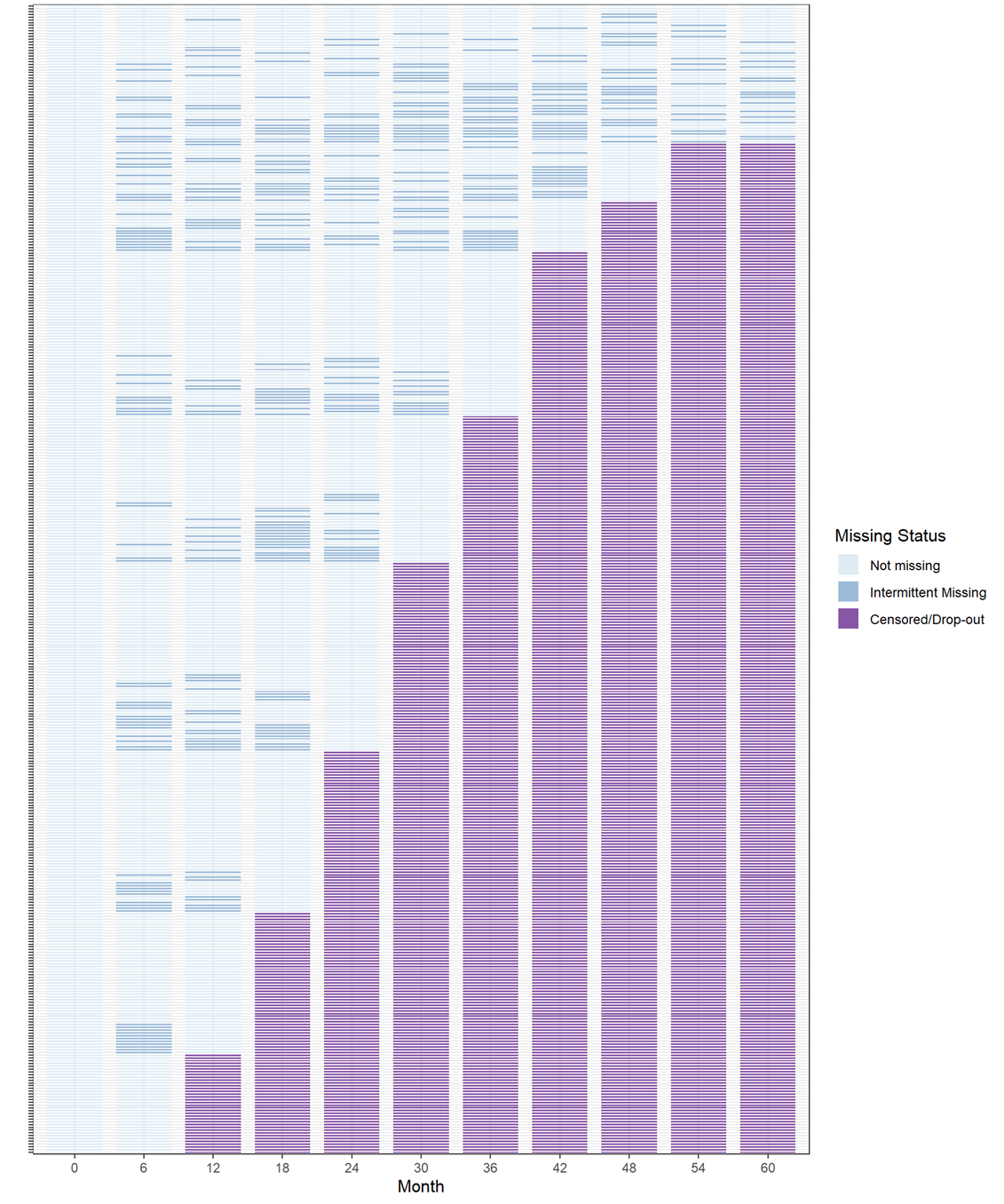

Supplement: S1 Fig — NSAA, North Star ambulatory assessment. NSAA assessments were assigned to the closest 6-month time point, drawing from the closest measured value within ±3 months of each time point. Each row represents an individual patient. (TIF) [file pone.0325736.s003.tif]

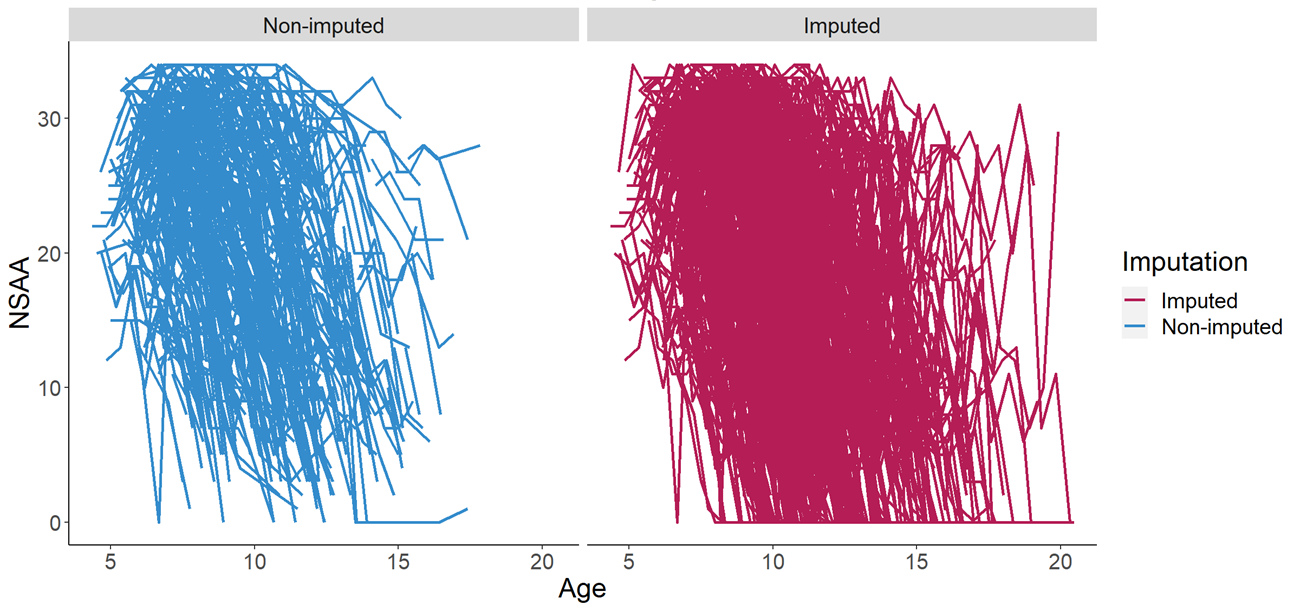

Supplement: S2 Fig — NSAA, North Star ambulatory assessment. (TIF) [file pone.0325736.s004.tif]

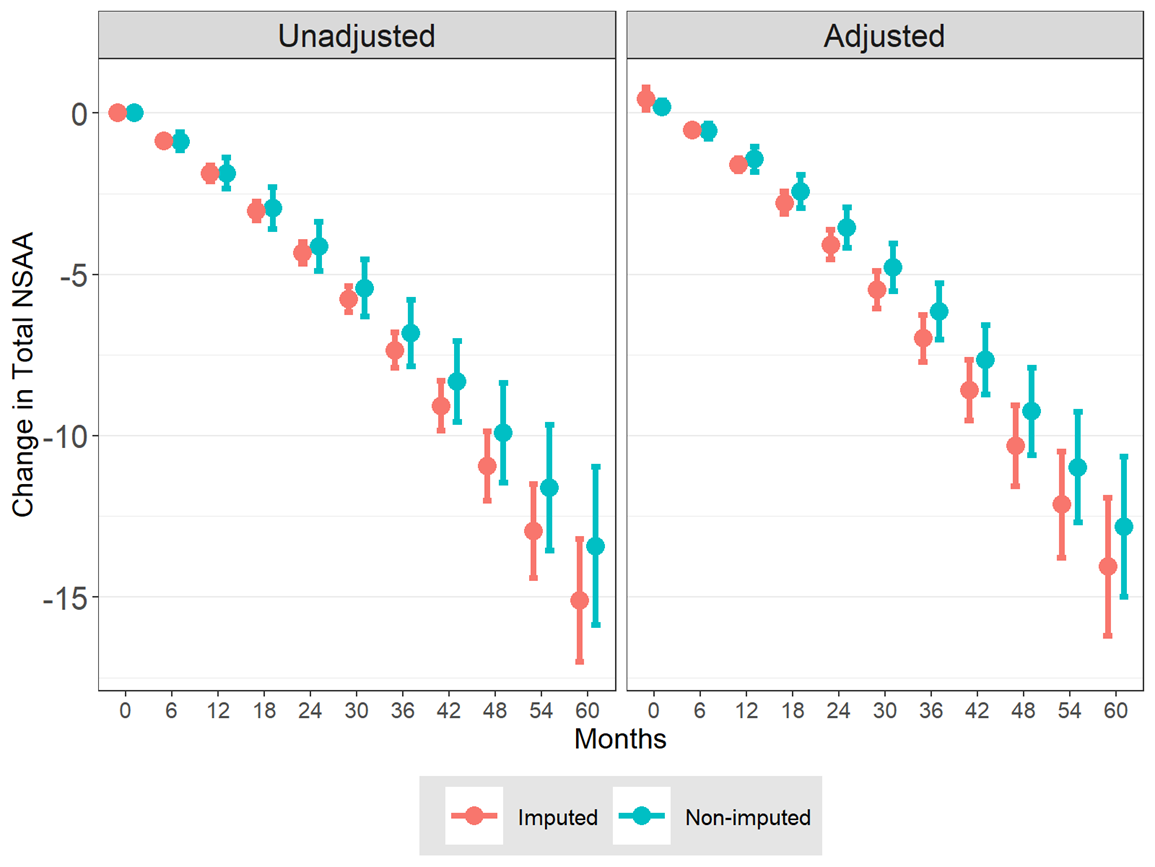

Supplement: S3 Fig — (TIF) [file pone.0325736.s009.tif]

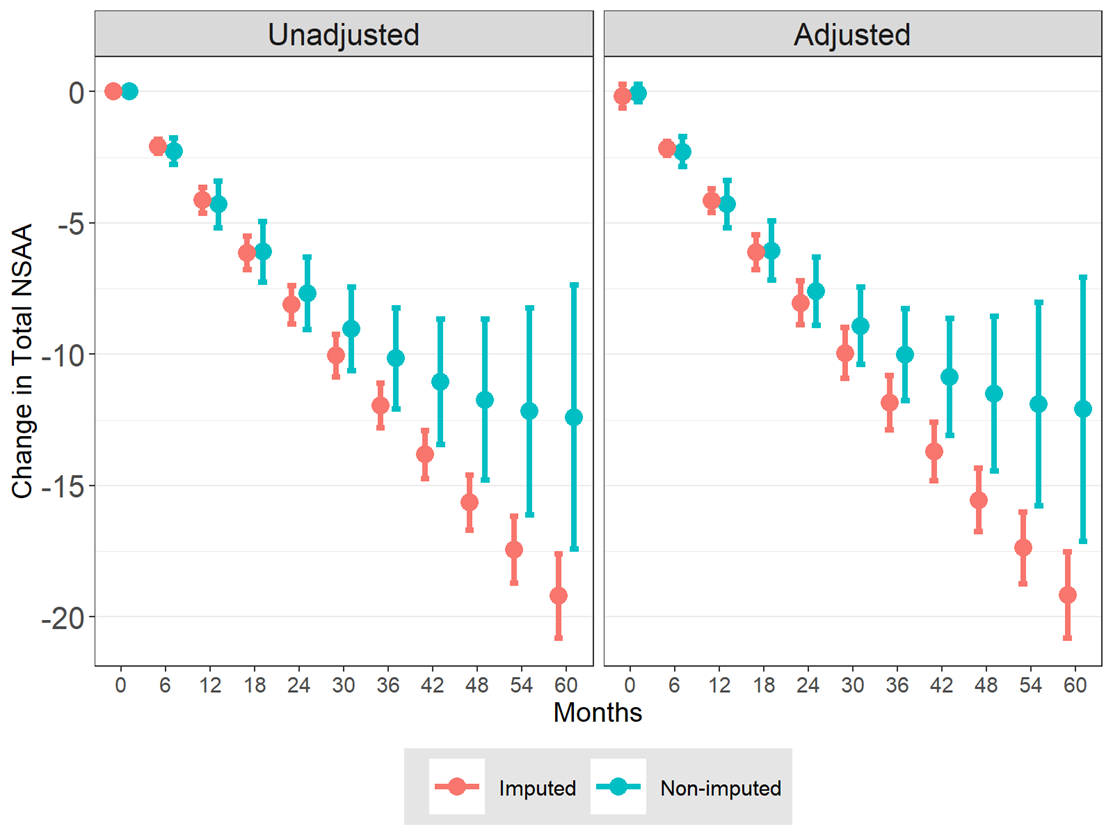

Supplement: S4 Fig — (TIF) [file pone.0325736.s010.tif]

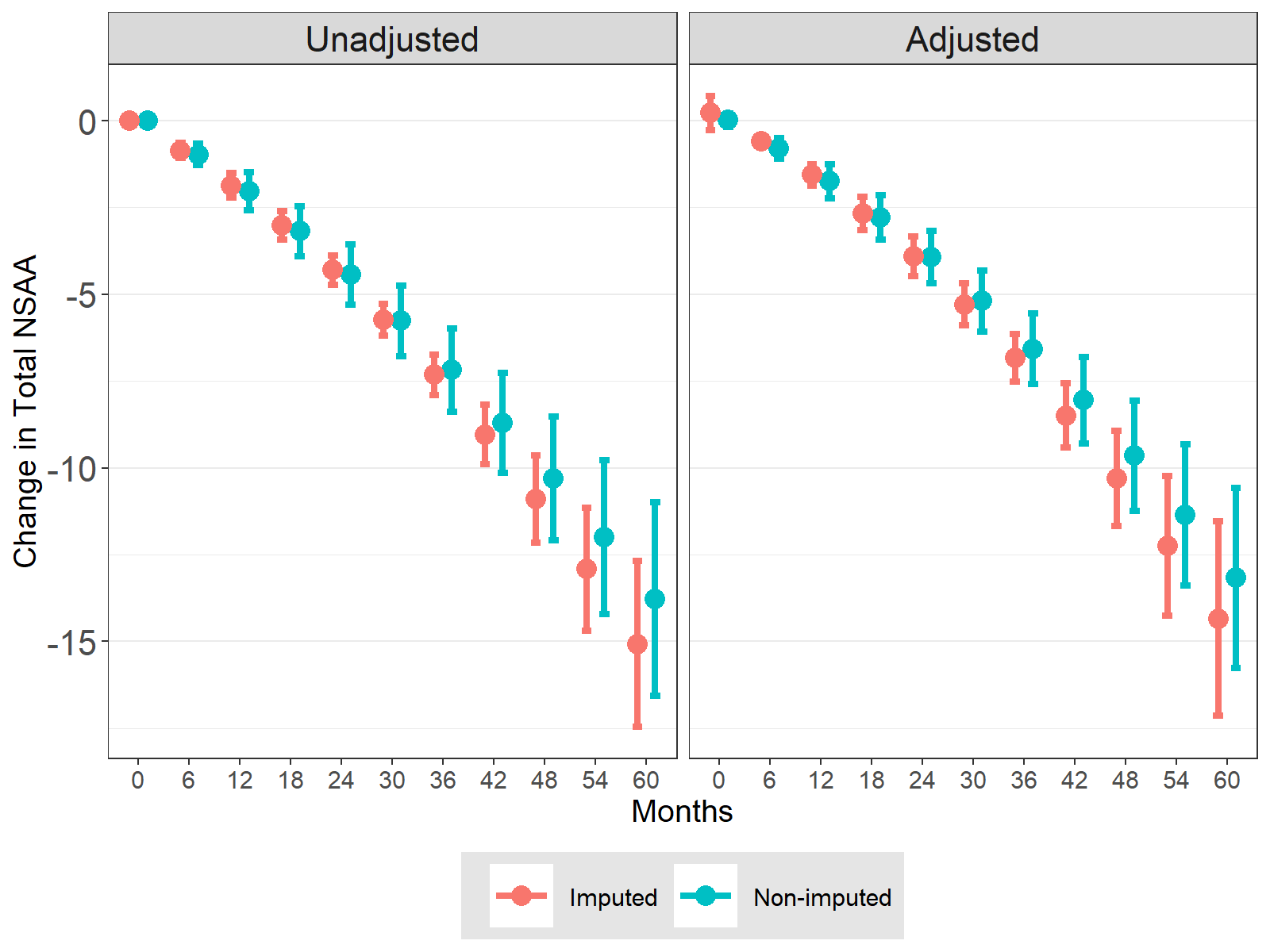

Supplement: S5 Fig — (TIF) [file pone.0325736.s011.tif]

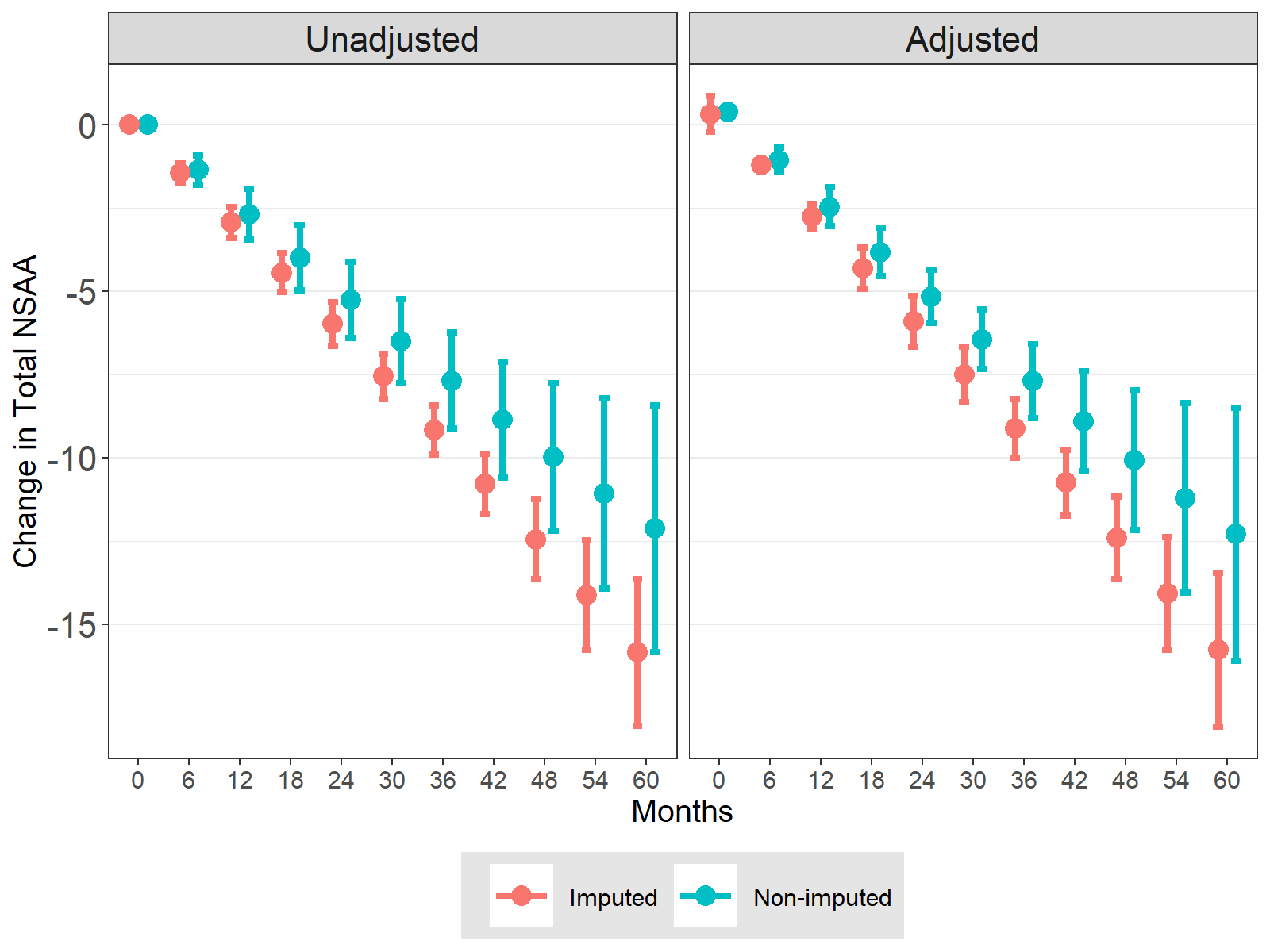

Supplement: S6 Fig — (TIF) [file pone.0325736.s012.tif]

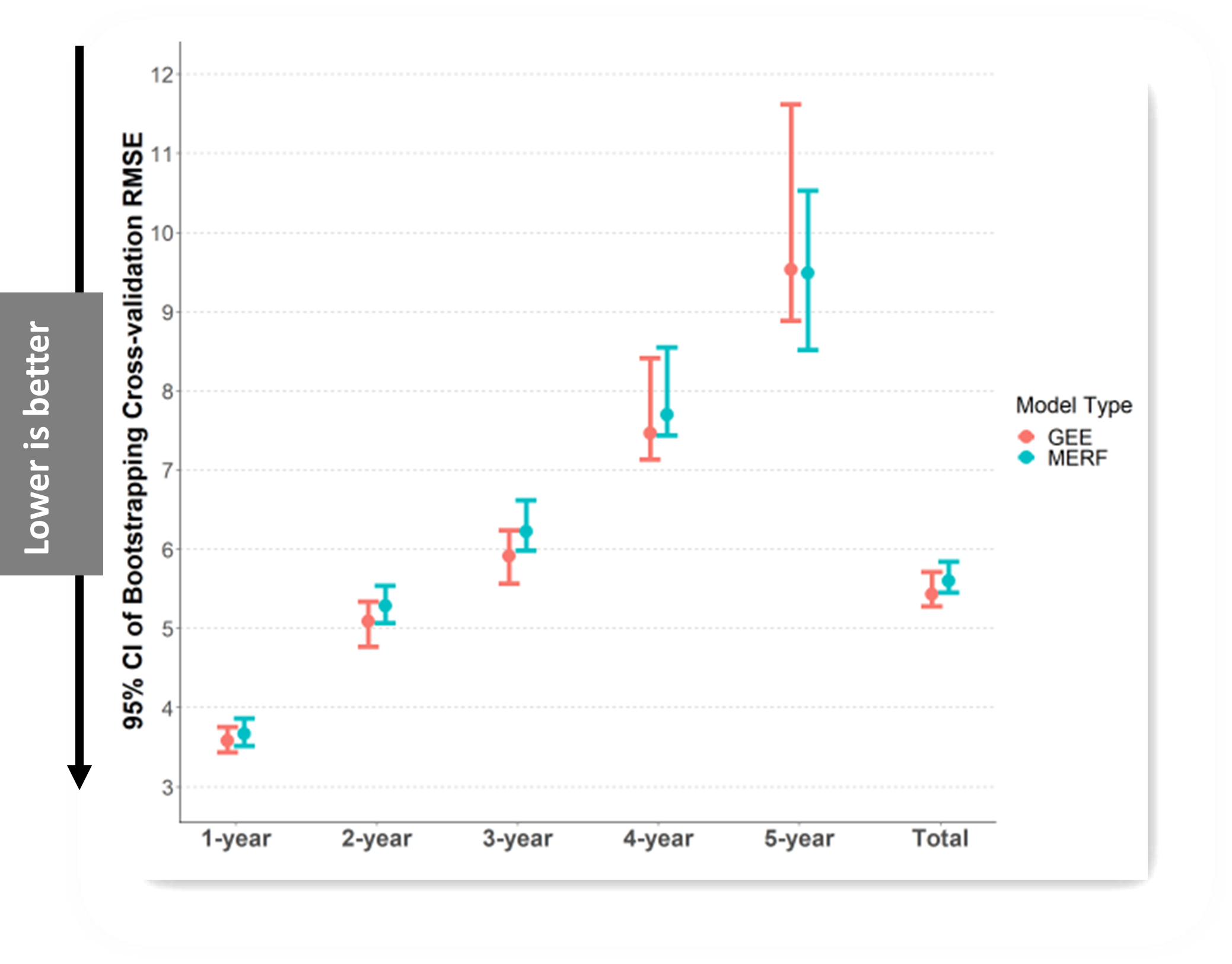

Supplement: S7 Fig — (TIF) [file pone.0325736.s013.tif]
