# Supplementary material for: Predicting trajectories of the north star ambulatory assessment total score in Duchenne muscular dystrophy
Source: PLoS One. 2025 Jun 27;20(6):e0325736. doi: 10.1371/journal.pone.0325736 (PMC12204569; doi:10.1371/journal.pone.0325736)
Supplement: S1 Table — (DOCX) [file pone.0325736.s005.docx]

S1 Table. Data Source Characteristics.

| PRO-DMD-01 (NCT01753804) | PRO-DMD-01 was a prospective observational study of disease progression in 269 individuals with DMD from 16 centers worldwide. Data were provided by CureDuchenne, a 501(3)c DMD patient foundation. Study assessments were scheduled every 6 months, and primarily occurred between 2012 and 2015. |
| --- | --- |
| Data source type | Prospectively collected natural history data |
| Study locations | 16 sites across USA, South America, and Europe |
| Key inclusion/exclusion criteria | Genetically proven DMD; age 3 to 18 years; willing and able to comply with protocol requirements; life expectancy of at least 3 years; able to give informed assent and/or consent in writing signed by the subject and/or parent(s)/legal guardian |
| Typical standard of care, including glucocorticoid use and physical therapy | At baseline 208 subjects (78%) were using steroids for DMD, mainly in a continuous (56.2%) or intermittent (15.4%) regimen, and 59 (22.1%) used none (mostly younger subjects). |
| Leuven | Data were collected from individuals with DMD during routine clinical practice at the Universitaire Ziekenhuizen pediatric neurology clinic in Leuven, Belgium. The database available for the present study included 155 individuals with clinic visits occurring primarily from 2011-2016. Clinic visits occurred approximately every 6  months. |
| Data source type | Curated RWD from individuals with DMD from routine clinical practice at the Universitaire Ziekenhuizen pediatric neurology clinic in Leuven, Belgium |
| Study locations | 1 center in Belgium |
| Key inclusion/exclusion criteria | Genetically proven DMD; aged 4.5 to 17.5 years; no severe cognitive or behavioral disorder impairing compliance |
| Typical standard of care, including glucocorticoid use and physical therapy | Glucocorticoid usually prescribed from age of 4 to 6 years onwards; 90% received 0.90 mg/kg daily deflazacort; physical therapy advice for prevention of contractures |
| iMDEX (NCT02780492) | iMDEX was a natural history study in 87 individuals with DMD from several centers in Europe. The iMDEX natural history study was funded by the Association Française contre les Myopathies. Study assessments occurred approximately every 6 months, and primarily occurred between 2013-2017. |
| Data source type | Prospective, longitudinal, multicenter observational study at neuromuscular centers |
| Study locations | 5 centers in Europe |
| Key inclusion/exclusion criteria | Diagnosis of DMD documented by MLPA or a standard genetic test for the disorder, genotypically confirmed to have an out-of-frame deletion(s) that could be corrected by skipping exon 51 or 53 or 45 or 44 or 46 or 50 or 52; ambulant children from 5 years old and teenagers with DMD; ability to walk independently for at least 75 meters in 6 minutes at recruitment; standard of care for DMD as recommended by the NorthStar UK and TREAT-NMD (i.e., on glucocorticoids treatment); sufficiently preserved pulmonary function (FVC >30%) and absence of symptoms of cardiac failure |
| Typical standard of care, including glucocorticoid use and physical therapy |  |
| North Star UK | The NSUK database contains clinical data for more than 500 ambulant individuals with DMD who were treated according to current standards of care in the UK. Data were collected from 24 pediatric neuromuscular centers in the NorthStar clinical network. Clinic visits occurred approximately every 6 months and, in the data used for the present study, occurred primarily between 2005 and the present. Genotyping was based on  MLPA for deletions and duplications, with sequencing only when no alterations were detected with MLPA. |
| Data source type | Prospective natural history study from specialist neuromuscular centers in the United Kingdom |
| Study locations | 24 centers in the United Kingdom |
| Key inclusion/exclusion criteria | DMD diagnosis confirmed by genetic testing and/or a muscle biopsy |
| Typical standard of care, including glucocorticoid use and physical therapy | Treated according to current standards of care in the UK |

DMD, Duchenne muscular dystrophy; MLPA, Multiplex Ligation-dependent Probe Amplification; NSAA, North Star ambulatory assessment; RWD, real-world data.
