# Supplementary material for: Predicting trajectories of the north star ambulatory assessment total score in Duchenne muscular dystrophy
Source: PLoS One. 2025 Jun 27;20(6):e0325736. doi: 10.1371/journal.pone.0325736 (PMC12204569; doi:10.1371/journal.pone.0325736)
Supplement: S2 Table — (DOCX) [file pone.0325736.s006.docx]

S2 Table. Exploratory Model Specifications.

| Predictors^a^ | Models | | | | | | | |
| --- | --- | --- | --- | --- | --- | --- | --- | --- |
|  | M11 | M12 | M13 | M14 | M15 | M16 | M17 | M18 |
| M10 specifications^b^ | ✓ | ✓ | ✓ | ✓ |  | ✓ |  | ✓ |
| Genotype classes | ✓ |  |  |  |  |  |  |  |
| Steroid regimen |  | ✓ |  |  |  |  |  |  |
| Data source |  |  | ✓ |  |  |  |  |  |
| Calendar year |  |  |  | ✓ |  |  |  |  |
| Cubic time |  |  |  |  | ✓ | ✓ |  |  |
| Piecewise linear time with knots at year 1, 2, 3, 4 |  |  |  |  |  |  | ✓ | ✓ |

10MWR, 10-meter walk/run; BMI, body mass index; M, model; NSAA, North Star Ambulatory Assessment; RFF, rise from floor.

^a^ Check marks indicate that the variable was included in the model.

^b^ M10 specifications can be found in Table 1 and includes baseline age, quadratic age, NSAA total score, 10MWR velocity, RFF velocity, steroid type, height, weight, and BMI.
