# Supplementary material for: Predicting trajectories of the north star ambulatory assessment total score in Duchenne muscular dystrophy
Source: PLoS One. 2025 Jun 27;20(6):e0325736. doi: 10.1371/journal.pone.0325736 (PMC12204569; doi:10.1371/journal.pone.0325736)
Supplement: S3 Table — (DOCX) [file pone.0325736.s007.docx]

**S3 Table. Exploratory Model Predictive Performance.**

|  | **1-year (N = 379)^a^** | | **2-year (N = 327)^a^** | | **3-year (N = 214)^a^** | | **4-year (N = 115)^a^** | | **5-year (N = 50)^a^** | | **Total (N = 416)** | |
| --- | --- | --- | --- | --- | --- | --- | --- | --- | --- | --- | --- | --- |
| **Model^b^** | RMSE | CV-RMSE | RMSE | CV-RMSE | RMSE | CV-RMSE | RMSE | CV-RMSE | RMSE | CV-RMSE | RMSE | CV-RMSE |
| M10 (Core) | 3.55 | 3.58 | 4.96 | 5.09 | 5.69 | 5.91 | 7.21 | 7.47 | 8.61 | 9.53 | 5.20 | 5.43 |
| **M11^c^** | 3.58 | 3.66 | 4.97 | 5.21 | 5.64 | 6.03 | 7.11 | 7.81 | 7.95 | 10.24 | 5.14 | 5.61 |
| **M12^d^** | 3.63 | 3.68 | 4.78 | 4.93 | 5.83 | 6.12 | 7.53 | 7.88 | 8.57 | 9.82 | 5.17 | 5.47 |
| **M13** | 3.56 | 3.62 | 4.95 | 5.12 | 5.65 | 5.98 | 6.95 | 7.39 | 8.24 | 9.23 | 5.13 | 5.42 |
| **M14^e^** | 3.55 | 3.61 | 4.95 | 5.11 | 5.68 | 5.92 | 7.14 | 7.46 | 8.35 | 9.50 | 5.17 | 5.44 |
| **M15** | 3.94 | 3.95 | 5.80 | 5.84 | 7.18 | 7.19 | 9.02 | 8.99 | 9.49 | 9.54 | 6.19 | 6.23 |
| **M16** | 3.51 | 3.60 | 4.95 | 5.14 | 5.71 | 5.94 | 7.21 | 7.48 | 8.43 | 9.87 | 5.18 | 5.49 |
| **M17** | 3.93 | 3.94 | 5.80 | 5.84 | 7.16 | 7.17 | 8.94 | 8.93 | 9.51 | 9.59 | 6.18 | 6.22 |
| **M18** | 3.52 | 3.59 | 4.95 | 5.11 | 5.72 | 5.99 | 7.09 | 7.52 | 8.44 | 10.15 | 5.17 | 5.52 |

CV-RMSE, cross-validated root mean squared error; M, model; RMSE, root mean squared error.

^a^ “N” corresponds to the maximum number of patients with available data at each time point. Some models include some missing data due to insufficient data for some variables included in those models.

^b^ Predictors included in each model can be found in Table S2.

^c^ Number of patients included in model 11 was 369, 320, 211, 111, 48, and 404 at years 1, 2, 3, 4, 5, and during the full follow up time, respectively. This was due to missing data for genotype classes.

^d^ Number of patients included in model 12 was 330, 288, 185, 93, 40, and 406 at years 1, 2, 3, 4, 5, and during the full follow up time, respectively. This was due to missing data for steroid regimen.

^e^ Number of patients included in model 14 was 378, 326, 213, 115, 50, and 415 at years 1, 2, 3, 4, 5, and during the full follow up time, respectively. This was due to missing data for calendar year.
