# Supplementary material for: Predicting trajectories of the north star ambulatory assessment total score in Duchenne muscular dystrophy
Source: PLoS One. 2025 Jun 27;20(6):e0325736. doi: 10.1371/journal.pone.0325736 (PMC12204569; doi:10.1371/journal.pone.0325736)
Supplement: S4 Table — (DOCX) [file pone.0325736.s008.docx]

**S4 Table. Fitted Models Including Steroid Regimen (Daily vs. Other) and Genotype Class.**

| **Baseline characteristics^a^** | **Model 11**  **Coefficient [95% CI]** | **Model 12**  **Coefficient [95%CI]** |
| --- | --- | --- |
| **Linear time × linear age** | −2.05 [−3.94, −0.16] | −1.79 [−3.66, 0.09] |
| **Quadratic time × linear age** | 0.10 [−0.51, 0.71] | 0.02 [−0.56, 0.61] |
| **Linear time × quadratic age** | 0.09 [−0.01, 0.19] | 0.08 [−0.02, 0.18] |
| **Quadratic time × quadratic age** | 0.00 [−0.04, 0.03] | 0.00 [−0.03, 0.03] |
| **Linear time × NSAA** | −0.17 [−0.31, −0.02] | −0.19 [−0.33, −0.05] |
| **Quadratic time × NSAA** | 0.02 [−0.02, 0.07] | 0.03 [−0.01, 0.07] |
| **Linear time × velocity RFF** | 13.11 [5.90, 20.31] | 16.68 [10.21, 23.14] |
| **Quadratic time × velocity RFF** | −1.80 [−3.98, 0.38] | −3.17 [−4.95, −1.40] |
| **Linear time × velocity 10MWR** | 1.95 [0.41, 3.49] | 1.65 [0.02, 3.27] |
| **Quadratic time × velocity 10MWR** | −0.37 [−0.84, 0.09] | −0.30 [−0.79, 0.19] |
| **Linear time × steroid use** | −0.98 [−2.39, 0.42] | −0.66 [−2.09, 0.78] |
| **Quadratic time × steroid use** | −0.10 [−0.59, 0.40] | −0.24 [−0.74, 0.26] |
| **Linear time × height** | −0.32 [−0.57, −0.06] | −0.31 [−0.56, −0.05] |
| **Quadratic time × height** | 0.03 [−0.05, 0.11] | 0.03 [−0.05, 0.11] |
| **Linear time × weight** | 0.50 [−0.03, 1.04] | 0.50 [−0.03, 1.02] |
| **Quadratic time × weight** | −0.02 [−0.19, 0.15] | −0.03 [−0.19, 0.14] |
| **Linear time × BMI** | −0.72 [−1.58, 0.14] | −0.64 [−1.48, 0.20] |
| **Quadratic time × BMI** | 0.03 [−0.22, 0.29] | 0.02 [−0.23, 0.27] |
| **Linear time × skip 44** | −2.03 [−4.89, 0.83] |  |
| **Quadratic time × skip 44** | 1.35 [0.21, 2.49] |  |
| **Linear time × skip 45** | −1.38 [−4.37, 1.61] |  |
| **Quadratic time × skip 45** | 0.95 [−0.26, 2.15] |  |
| **Linear time × skip 51** | −1.19 [−4.15, 1.77] |  |
| **Quadratic time × skip 51** | 0.88 [−0.26, 2.01] |  |
| **Linear time × skip 53** | −0.98 [−3.88, 1.93] |  |
| **Quadratic time × skip 53** | 0.76 [−0.37, 1.89] |  |
| **Linear time × other skip amenable** | −0.93 [−3.85, 2.00] |  |
| **Quadratic time × other skip amenable** | 0.84 [−0.32, 2.00] |  |
| **Linear time × all other genotypes** | −1.53 [−4.17, 1.11] |  |
| **Quadratic time × all other genotypes** | 0.98 [−0.11, 2.07] |  |
| **Linear time × daily steroids** |  | 1.12 [−0.31, 2.55] |
| **Quadratic time × daily steroids** |  | −0.46 [−0.89, −0.03] |

BMI, body mass index; NSAA, North Star ambulatory assessment; RFF, rise from floor; 10MWR, 10-meter walk/run.

^a^Time and age are measured in years; RFF velocity is calculated as 1 / completion time; 10MWR velocity is measured in meters / second; height is measured in cm; weight is measured in kg; BMI is measured in kg/m^2^.
